# 获得性凝血因子缺乏症的发病机制、诊断与治疗

**DOI:** 10.3760/cma.j.issn.0253-2727.2023.11.014

**Published:** 2023-11

**Authors:** 丹丹 余, 葳 刘, 磊 张

**Affiliations:** 中国医学科学院血液病医院（中国医学科学院血液学研究所），实验血液学国家重点实验室，国家血液系统疾病临床医学研究中心，细胞生态海河实验室，天津市血液病基因治疗研究重点实验室，中国医学科学院血液病基因治疗重点实验室，天津 300020 State Key Laboratory of Experimental Hematology, National Clinical Research Center for Blood Diseases, Haihe Laboratory of Cell Ecosystem, Institute of Hematology & Blood Diseases Hospital, Chinese Academy of Medical Sciences & Peking Union Medical College, Tianjin Key Laboratory of Gene Therapy for Blood Diseases, CAMS Key Laboratory of Gene Therapy for Blood Diseases, Tianjin 300020, China; 2 天津医学健康研究院，天津 301600 Tianjin Institutes of Health Science, Tianjin 301600, China

获得性凝血因子缺乏症是一组由于各种原因导致患者血浆中凝血因子活性下降引起的获得性出血性疾病，包括获得性凝血因子I（FI）（纤维蛋白原）、FII（凝血酶原）、FV、FVII、FVIII、FIX、FX、FXI、FXII、FXIII和血管性血友病因子（vWF）缺乏。其发病机制主要包括凝血因子合成减少（如肝脏疾病、维生素K缺乏等）、消耗或破坏增多［如弥漫性血管内凝血（DIC）］及产生抗凝血因子抗体（自身免疫性疾病、恶性肿瘤等）等[1]。其中，由患者体内产生抗凝血因子抗体引起的获得性凝血因子缺乏症较为罕见，且临床表现异质性较大，目前对其诊断及治疗较为复杂。本文从发病机制、临床特点、诊断及治疗等方面对由抗凝血因子抗体引起的获得性凝血因子缺乏症进行综述。

一、发病机制及临床表现

抗凝血因子抗体包括同种抗体（如血友病A/B患者输注FⅧ/FⅨ后产生的抗FⅧ/FⅨ抗体）和自身抗体（自身免疫性疾病、恶性肿瘤、感染等引起的抗体）。获得性凝血因子缺乏症通常由后者引起，包括与凝血因子特异性结合后影响其活性的中和性抗体及加速其清除的非中和性抗体[2]（表1）。

**表1:** 获得性凝血因子缺乏症的分类

抗体类型及作用机制	相关疾病
FⅠ（抑制活性或加速清除）	特发性、妊娠、自身免疫性疾病、恶性肿瘤等
FⅡ（抑制活性或加速清除）	特发性、牛凝血酶、自身免疫性疾病、恶性肿瘤、感染等
FⅤ（抑制活性）	牛凝血酶、抗生素、恶性肿瘤、自身免疫性疾病等
FⅦ（抑制活性或加速清除）	特发性、恶性肿瘤、自身免疫性疾病、感染、妊娠等
FⅧ（抑制活性）	特发性、自身免疫性疾病、恶性肿瘤、感染、妊娠等
FⅨ（抑制活性）	特发性、自身免疫性疾病、感染、产后等
FⅩ（抑制活性或加速清除）	特发性、恶性肿瘤、自身免疫性疾病、抗生素等
FⅪ（抑制活性或加速清除）	自身免疫性疾病、恶性肿瘤、感染、妊娠等
FⅫ（抑制活性）	自身免疫性疾病、恶性重量、感染等
FXIII（抑制活性或加速清除）	自身免疫性疾病、恶性肿瘤、感染等
vWF（抑制活性或加速清除）	血液系统疾病（MM、MGUS及WM等）、自身免疫性疾病、其他恶性肿瘤等

注 FⅠ：凝血因子Ⅰ；FⅡ：凝血因子Ⅱ；FⅤ：凝血因子Ⅴ；FⅦ：凝血因子Ⅻ；FⅧ：凝血因子Ⅷ；FⅨ：凝血因子Ⅸ；FⅩ：凝血因子Ⅹ；FⅪ：凝血因子Ⅺ；FⅫ：凝血因子Ⅻ；FXIII：凝血因子XIII；vWF：血管性血友病因子；MM：多发性骨髓瘤；MGUS：单克隆免疫球蛋白血症；WM：华氏巨球蛋白血症

1. 获得性FⅧ缺乏症（获得性血友病A，AHA）：FⅧ是一种主要由肝脏和内皮细胞合成的糖蛋白，与vWF结合形成复合物循环于血液中，在内源性凝血途径的中间阶段发挥促凝作用。经凝血酶及其他蛋白酶水解激活后的FⅧ（FⅧa）在活化的FⅨ（FⅨa）、钙离子及磷脂存在的情况下催化FⅩ转化为活化的FⅩ（FⅩa）[3]。抗FⅧ抗体多为多克隆IgG抗体，主要包括IgG1和IgG4亚类，也有部分为IgA及IgM抗体。其与FⅧ分子的活性区域结合干扰FⅧ与FⅨa、磷脂及vWF的相互作用从而影响其促凝活性。该抗体是一种时间-温度依赖性抗体，即其对FⅧ活性的抑制作用随时间和温度而增强[4]。由抗FⅧ抗体引起的AHA是最常见的获得性凝血因子缺乏，发病率约为1.5/100万。该病可发生于男女各年龄段，两个发病高峰分别为育龄女性的围产期及60岁以上人群，儿童罕见。约一半的患者可以发现潜在病因，如自身免疫性疾病、恶性肿瘤、药物、感染等，少数患者发生于妊娠期或产后1年内[5]。约10％的AHA患者无出血表现，而大部分出血患者通常表现为严重出血（>65％）。最常见的出血表现是皮下出血，其次是肌肉出血，关节出血很少见[6]。FⅧ活性及FⅧ抑制物水平与出血严重程度无明显相关性，但可能会影响患者的止血疗效以及使患者达到完全缓解（CR）的时间延长[7]。

2. 获得性vWF缺乏症（获得性血管性血友病，AVWD）：vWF是一种主要由内皮细胞和巨核细胞合成的糖蛋白，以多聚体形式在血浆中循环，其作用是与血浆中FⅧ结合以防止其降解，以及介导血小板在血管损伤部位聚集形成血小板血栓。因此，各种原因引起的vWF缺乏可引起机体异常出血[8]。抗vWF抗体多为IgG，也有少部分IgM和IgA。抗vWF抗体可能通过与vWF结合抑制其瑞斯托霉素辅因子活性或抑制vWF与胶原的结合能力从而影响其止血功能；也可能与vWF形成免疫复合物加速vWF从循环中清除[9]。该抗体的产生通常与单克隆免疫球蛋白血症（MGUS）、多发性骨髓瘤（MM）和华氏巨球蛋白血症（WM）等血液系统疾病有关，部分患者也可由其他恶性肿瘤、自身免疫性疾病等引起[10]–[11]。AVWD患者的中位年龄为60岁，其出血症状可表现为自发性皮肤黏膜出血（皮肤瘀斑、鼻出血、月经过多、胃肠道出血或血尿等）或创伤及手术后出血过多[12]。

3. 获得性FⅠ缺乏症（AFⅠD）：纤维蛋白原是血液中含量最高的凝血因子。组织和血管损伤时，它可以与血小板结合促使血小板聚集形成血小板血栓，也可以在凝血酶的作用下转化为纤维蛋白，进而转化为纤维蛋白血凝块，起到止血的作用[13]。抗FⅠ抗体极为罕见，目前报道的病例主要与围产期、恶性肿瘤、自身免疫性疾病等有关。也有特发性及少数由其他疾病引起的抗FⅠ抗体相关病例报道。围产期及自身免疫性疾病患者体内激活的自身抗体可能与纤维蛋白原的异常聚合有关；多发性骨髓瘤（MM）患者中增多的免疫球蛋白可能导致纤维蛋白原的异常聚合也可引起纤维蛋白原的清除加快[14]。获得性FⅠ缺乏患者可能无明显症状，也可能表现为出血或血栓形成。其临床表现的异质性取决于潜在相关病因以及其他凝血因子是否受到影响[15]。

4. 获得性FⅡ缺乏症（AFⅡD）：凝血酶原是一种维生素K依赖性凝血因子，在血液凝固的最后阶段，被FⅩa激活形成凝血酶，进而使纤维蛋白原转变为纤维蛋白。此外，凝血酶还可以通过诱导血小板聚集、激活及水解其他介质参与凝血与抗凝过程。因此，凝血酶原缺乏或结构异常会导致凝血机制的异常[16]。抗凝血酶原抗体是一种抗磷脂抗体，大部分以低亲和力与凝血酶原结合，在抗磷脂抗体综合征（APS）中可能与血栓形成相关[17]。而在与狼疮抗凝物（LA）同时存在时，抗凝血酶原抗体表现出与凝血酶原的高度亲和力，与之结合形成复合物，加速其在血循环中的清除，引起低凝血酶原血症，称为狼疮抗凝物-低凝血酶原血综合征（LAHS）[18]。一些非中和性抗凝血酶原抗体也可在无LA的情况下出现，在部分患者中的抗凝血酶原抗体也可表现为对FII的促凝活性起抑制作用[19]。在过去的外科手术中使用牛凝血酶的患者体内可能产生抗凝血酶抗体，可能导致严重出血，其原因可能与抑制纤维蛋白形成、抑制血小板活化等有关[20]。LAHS主要与自身免疫性疾病（系统性红斑狼疮等）相关，也可见于感染及淋巴瘤等患者。LAHS更常见于女性，中位发病年龄为13～22岁。约89％的患者表现为出血，13％的患者表现为血栓形成。出血表现以鼻出血和皮肤出血较多见，女性患者也可表现为月经过多[18]。

5. 获得性FⅤ缺乏症（AFⅤD）：FⅤ是一种主要由肝脏和巨核细胞合成的糖蛋白，以非活性状态循环于血浆中（占80％）和储存于血小板中（占20％）。经激活的FⅤ（FⅤa）作为辅因子参与FⅩa催化凝血酶原形成凝血酶发挥促凝作用，被活化蛋白C（APC）裂解后的FⅤ也可通过灭活FⅧ发挥抗凝作用[21]。由抗FⅤ抗体引起的获得性FⅤ包括抗FⅤ自身抗体及外科手术中使用牛凝血酶制剂后产生的抗牛凝血酶的交叉抗体。抗FⅤ自身抗体常继发于使用抗生素类药物、自身免疫性疾病、恶性肿瘤、感染等。在重组人凝血酶代替了牛凝血酶在手术中用药之后，抗FⅤ自身抗体为引起获得性FⅤ缺乏的主要原因[22]。抗FⅤ自身抗体通常为IgG型抗体，与FⅤ轻链C2结构域（为与活化血小板或内皮细胞暴露的促凝的磷脂结合表位）结合从而抑制其促凝活性。该病常见于老年患者（>60岁），无明显性别差异。其临床表现差异较大，患者可无出血表现，也可出现危及生命的严重出血（如颅内出血），有的患者也可出现血栓。出血表现的差异性可能与FⅤ抑制物是否影响血小板中的FⅤ以及其识别FⅤ的不同表位有关[23]。出血症状常累及多个部位，最常见的是泌尿系出血、消化道出血及手术部位出血，脑出血的发生率较低但死亡率较高（21％）。其出血倾向与FⅤ活性及FⅤ抑制物水平无明显相关性[1],[24]。

6. 获得性FⅦ缺乏症（AFⅦD）：FⅦ是一种维生素K依赖性凝血因子，也是外源性凝血途径的启动因子。机体损伤后，组织因子（TF）释放入血，激活FⅦ形成FⅦa，FⅦa与TF结合形成复合物进一步激活FⅩ及FⅨ参与凝血反应[3]。抗FⅦ抗体极为罕见，既往报道的病例主要与恶性肿瘤、自身免疫性疾病及药物暴露等相关[25]。抗FⅦ抗体为抗FⅦ分子轻链的IgG自身抗体，该抗体可能干扰FⅦ与磷脂膜或TF的结合从而抑制其促凝活性。有的患者体内也发现存在加速FⅦ清除的非中和性抗FⅦ抗体[26]。该病患者可无出血表现，也可发生危及生命的严重出血。出血风险与FⅦ活性及抑制物水平无明显相关性[25]。

7. 获得性FⅨ缺乏症（获得性血友病B，AHB）：FⅨ是一种维生素K依赖性凝血因子。经TF-FⅦa复合物或XⅠa活化后的FⅨ（FⅨa）和FⅧa一起促进FⅩ的激活[16]。抗FⅨ抗体与抗FⅧ抗体类似，通常为多克隆IgG1和IgG4型的中和性抗体[3]。与抗FⅧ抗体相比，抗FⅨ抗体极其罕见，可能与FⅨ分子量小、免疫原性低有关[27]。既往报道的由抗FⅨ抗体引起的AHB主要与自身免疫性疾病、感染及产后等有关。该病患者可表现为轻度出血，也可发生危及生命的严重出血。出血常发生在皮肤黏膜以及肌肉等部位[28]。

8. 获得性FⅩ缺乏症（AFⅩD）：FⅩ也是一种维生素K依赖性凝血因子，是共同凝血途径中的一个重要酶原。经FⅦa或FⅨa激活的FⅩ在FⅤa、钙离子和磷脂的存在下将凝血酶原裂解为凝血酶[16]。淀粉样变性为获得性FⅩ缺乏（AFⅩD）最常见的病因，其发病机制可能是由于高负荷淀粉样纤维在网状内皮系统中吸附FⅩ从而引起FⅩ缺乏[29]。而由抗FⅩ抗体引起的AFⅩD仅在少数与淀粉样变性无关的病例中有报道，包括影响FⅩ促凝活性的中和性抗体和加快FⅩ清除的非中和性抗体。主要与恶性肿瘤、自身免疫性疾病和抗生素类药物使用相关[30]。AFⅩD患者的出血表现大多为有创检查后出血、颅内出血、关节肌肉出血等。其出血严重程度与FⅩ活性无明显相关性，但FⅩ活性<10％的患者有更高的自发性出血风险[1]。

9. 获得性FⅪ缺乏症（AFⅪD）：FⅪ是内源性凝血途径中的一个重要凝血因子，经FⅦa激活后的FⅪa进一步激活FⅨ，再通过一系列的酶促反应引导凝血酶形成。目前也有研究表明FⅪ可直接被凝血酶激活，并可以通过外源性凝血途径激活FⅦ[31]。抗FⅪ抗体很少见，包括影响FⅪ活性的中和性抗体及加速其清除的非中和性抗体。其发生通常与自身免疫性疾病（以系统性红斑狼疮为主）、恶性肿瘤、妊娠及感染有关[32]。该病通常不会引起自发性出血，但可能在手术、创伤或分娩时引起严重出血。有的患者还可表现为血栓形成[33]。

10. 获得性FⅫ缺乏症（AFⅫD）：在经典的凝血瀑布学说中，FⅫ是启动内源性凝血途径的初始因子，还参与激活纤维蛋白溶解系统、活化补体及炎症反应。目前认为FⅫ在炎症反应和血栓形成中发挥的作用更重要[34]。抗FⅫ抗体较罕见，其发病机制尚未明确，通常与自身免疫性疾病、恶性肿瘤、感染有关。FⅫ缺乏可引起活化部分凝血活酶时间（APTT）延长，但不会引起异常出血表现。相反，系统性红斑狼疮患者有血栓形成的风险，可能与抗FⅫ抗体抑制FⅫ的纤溶活性有关[35]。

11. 获得性FXIII缺乏症（AFXIIID）：FXIII是一种纤维蛋白稳定因子，包含2个催化亚单位A和2个非催化亚单位B。经凝血酶活化的FXIII（FXIIIa）可以发挥交叉连接纤维蛋白单体以及抗纤维蛋白溶解和蛋白水解的作用，稳定凝块，有助于止血和伤口愈合[36]。抗FXIII抗体包括抗FXIII-A亚单位的中和性抗体和抗FXIII-B亚单位的非中和性抗体，以前者为主。抗FXIII-A亚单位抗体可能通过扰凝血酶对FXIII的激活或改变FXIII在纤维蛋白原上的结合位点抑制其活性[37]。由抗FXIII抗体引起的获得性FXIII缺乏症常与自身免疫性疾病、恶性肿瘤、感染等相关。主要表现为自发性肌肉或皮下出血、术后出血过多及伤口愈合延迟等，也可发生颅内出血（为其主要死亡原因）。患者出血情况与FXIII活性无明显相关性[38]。

二、实验室检查及诊断

获得性凝血因子缺乏症的实验室检查包括血常规、常规凝血功能筛查［APTT、凝血酶原时间（PT）、凝血酶时间（TT）、纤维蛋白原等］，APTT/PT混合血浆纠正试验，凝血因子活性检测及凝血抑制物定量检测（图1）[39]。对于有异常出血表现且既往无出血个人史及家族史的患者，应考虑此病，应逐步完善上述实验室检查以明确诊断。若APTT/PT延长，则进一步行混合血浆纠正试验初步筛查是否存在凝血因子抑制物，即将患者血浆和正常人血浆1∶1混合后再分别测定APTT/PT（即刻和37 °C孵育2 h），与正常血浆相比若延长5 s以上（或延长>15％）则为不能纠正[40]。若混合血浆APTT/PT不能被纠正，可考虑有凝血因子抑制物的存在，但狼疮抗凝物及抗凝剂（如肝素、口服抗凝药等）也可出现此结果，需注意鉴别。凝血因子活性检测若提示多个凝血因子同时缺乏可能由肝脏疾病、维生素K缺乏、DIC等情况引起，也可能由抑制物（如狼疮抗凝物或凝血因子抗体）的干扰引起，需注意鉴别。因FXIII缺乏不会引起APTT/PT延长，故对于有异常出血但常规凝血功能筛查阴性的患者，可考虑进行FXIII活性检测。通过往血凝块中加入浓缩尿素溶液，观察血凝块是否在24 h内溶解来对FXIII活性进行初步筛查（正常人为24 h内血凝块不溶解）。但该定性试验只有在FXIII活性降至0.5％～5％才会显示阳性，对轻中度FXIII缺乏可能不敏感。中和性FXIII抑制物的存在通过与等量正常血浆混合后重复该试验来确定[41]。中和性凝血因子抗体滴度主要通过Bethesda测定法或Nijmegen改良法进行检测（检测临界值为0.6 BU），非中和性凝血因子抗体可通过测定凝血因子回收率及半衰期分析间接证明其存在[42]。

**图1:**
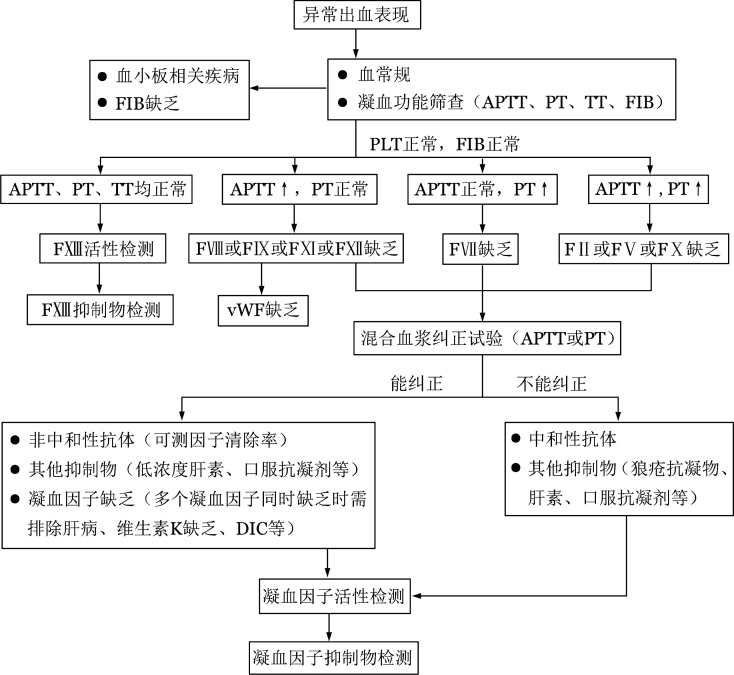
获得性凝血因子缺乏症的诊断流程 注 APTT：活化部分凝血活酶时间；PT：凝血酶原时间；TT：凝血酶时间；FIB：纤维蛋白原；PLT：血小板计数；FⅡ：凝血因子Ⅱ；FⅤ：凝血因子Ⅴ；FⅦ：凝血因子Ⅶ；FⅧ：凝血因子Ⅷ；FⅨ：凝血因子Ⅸ；FⅩ：凝血因子Ⅹ；FⅪ：凝血因子Ⅺ；FⅫ：凝血因子Ⅻ；FXIII：凝血因子XIII；vWF：血管性血友病因子；LA：狼疮抗凝物；DIC：弥散性血管内凝血

三、治疗

获得性凝血因子缺乏症的治疗主要包括预防出血和及时止血治疗以及抗体清除治疗。同时应积极去除诱因及治疗原发病。

1. 止血治疗：除AFⅫD外，获得性凝血因子缺乏症患者均应注意休息，避免外伤、手术及有创操作。获得性FⅫ缺乏症的患者没有出血风险，不需常规进行止血治疗，合并系统性红斑狼疮或恶性肿瘤的患者应酌情考虑抗凝治疗。止血治疗以控制急性出血为主，同时应注意血栓形成的风险。

由于AHA患者出血表现与因子活性及抑制物滴度的相关性通常较差，故止血治疗应根据患者的出血严重程度进行。因高滴度FⅧ抑制物的存在，选用旁路制剂重组活化人凝血因子Ⅶ（rFⅦa，90 µg/kg每2～3 h 1次）和活化凝血酶原复合物（aPCC）作为AHA患者的首选止血药物，同时应注意其血栓形成的风险[43]。因aPCC未在国内上市，故当无法使用rFⅦa时，可选凝血酶原复合物（PCC）150 U·kg^−1^·d^−1^进行止血治疗。使用旁路制剂治疗时应密切监测患者血小板数量、纤维蛋白原和D-二聚体，以评估患者血栓形成的风险。无法使用上述旁路制剂或疗效不佳且患者抑制物滴度较低（<5 BU/ml）时，可使用高剂量FⅧ（50～100 U/kg，根据实际FⅧ活性调整）替代治疗。此外，去氨加压素（DDVAP）、抗纤溶药物如氨甲环酸等可作为其辅助止血治疗。若上述方法均无效，可采用血浆置换或免疫吸附联合FⅧ替代治疗以控制紧急出血[6]。双特异性抗体艾美塞珠单抗（模拟FⅧ同时与FⅨ和FⅩ结合发挥作用）也逐渐被用于AHA患者，但由于其血药浓度达峰时间较长，目前只能将其用于预防出血而非紧急出血的治疗[44]。近日，FⅩ激活剂STSP-0601已在国内完成在血友病A/B伴高滴度抑制物（>5 BU/ml）患者中的Ⅰ期临床试验，证实了其在血友病A/B伴高滴度抑制物患者中的安全性及提高FⅩ活性及缩短APTT的有效性，未来有望成为获得性血友病患者新的止血药物选择[45]。

通过抑制抗凝系统如抗凝血酶、组织因子途径抑制物（TFPI）和蛋白C系统以控制出血成为一种新的止血治疗选择。一项Ⅰ期临床试验表明抗凝血酶抑制剂（ALN-AT3SC，Fitusiran）能够降低约80％的抗凝血酶活性，从而使凝血酶生成增加，以控制出血[46]。Fitusiran用于伴或不伴抑制物血友病患者的多项临床试验也正在进行（Clinicaltrials.gov：NCT02554773、NCT03754790、NCT03974113）。一项Concizumab（一种人源化抗TFPI单克隆抗体）预防性疗法的血友病伴抑制物患者发生需治疗的自发与创伤型出血的Ⅲ期临床试验显示，与没有接受预防性治疗的患者相比的年出血率降低86％（Clinicaltrials.gov：NCT04082429）。目前，通过抑制蛋白C系统进行止血还处于临床前研究阶段[47]。

AVWD患者的止血治疗方案应根据出血严重程度进行选择。小手术或中度出血可使用DDVAP、FⅧ/vWF浓缩物等，也可选用抗纤溶制剂如氨甲环酸辅助治疗；重度出血可使用rFⅦa旁路制剂；与单克隆丙种球蛋白病相关的AVWD可采用静脉注射免疫球蛋白（IVIg）0.4 g·kg^−1^·d^−1^×5 d或1.0 g·kg^−1^·d^−1^×2 d治疗，疗效可持续3～5周[12]。血浆置换和免疫抑制治疗（IST）对严重出血的患者可能有效。

其他罕见获得性凝血因子缺乏症患者的止血治疗应以凝血因子活性及其抑制物滴度为依据。对于某些抑制物滴度较低（<5 BU/ml）的患者，可以通过使用高剂量的相应缺乏因子浓缩剂来中和抗体从而提高凝血因子活性进行达到止血目的。我国目前只有单独的纤维蛋白原、FⅡ、FⅦ、FⅨ血浆源性或重组因子浓缩制剂。同时，应密切监测实际凝血因子水平，以便调整给药剂量及时间，确保止血疗效。对于无法获得单一因子浓缩制剂的患者，可使用含多因子的血浆源性制剂进行止血治疗。如对于AFⅩD患者，可选用PCC补充FⅩ，同时密切监测FⅩ活性。需注意，反复输注PCC可导致患者体内其他凝血因子增多，从而增加发生血栓事件的风险。对于AFXIIID患者，可选择冷沉淀进行替代治疗。新鲜冰冻血浆（FFP）中凝血因子浓度较低，往往需要输注大量的FFP才能达到止血水平，导致患者的循环负荷过载，因此，FFP一般不作为获得性凝血因子缺乏症患者的首选止血药物。然而，对于AFⅤD患者，FFP为其主要治疗选择。因血小板内也含有部分FⅤ，故AFⅤD患者也可输注血小板进行辅助止血治疗[24]。对于高抑制物滴度患者（≥5 BU/ml）或对上述制剂无效的患者可尝试超适应证用药（使用旁路制剂rFⅦa或aPCC），但需密切监测疗效及关注是否有血栓形成的风险[1]。

2. 抗体清除治疗：对于与恶性肿瘤、感染、药物及妊娠等疾病相关的获得性凝血因子缺乏症应积极去除诱因及治疗原发病，经处理后的部分患者体内的凝血因子抑制物可以消失、凝血因子活性可恢复正常[48]。对于经上述处理后凝血因子缺乏尚未得到完全纠正的患者以及同时患有自身免疫性疾病的患者或为原发性获得性凝血因子缺乏症的患者应进行IST。

AHA患者的一线IST方案包括：①糖皮质激素（泼尼松1 mg·kg^−1^·d^−1^）单药治疗；②联合环磷酰胺（1.5～2 mg·kg^−1^·d^−1^，最多6周）治疗；③利妥昔单抗（375 mg/m^2^ 每周1次，最多4次或100 mg/m^2^每周1次×4次）治疗。糖皮质激素疗程一般不超过6周，在患者获得缓解或达到6周后逐渐减量至停用。AHA患者一线治疗的CR率为60％～80％[48]–[49]。有研究表明，FⅧ活性<1％或FⅧ抑制物≥5 BU/ml的患者与FⅧ活性≥1％且FⅧ抑制物<5 BU/ml的患者相比，IST的CR率较低，达到CR的时间也较长[7],[49]。也有研究表明，糖皮质激素联合环磷酰胺或利妥昔单抗与单独使用糖皮质激素相比，起效更快，缓解率更高，但是感染等并发症的发生率也相应增加[5],[43],[49]。因此，2020年AHA国际治疗指南建议高滴度抑制物（≥5 BU/ml）或FⅧ严重缺乏（FⅧ活性<1％）的AHA患者可首选糖皮质激素联合环磷酰胺或利妥昔单抗治疗[6]。

若一线治疗3～5周疗效不佳应考虑二线治疗，既往单用糖皮质激素的患者加用环磷酰胺或利妥昔单抗治疗，既往已联合环磷酰胺或利妥昔单抗的患者换用未使用过的药物（环磷酰胺或利妥昔单抗）。若一、二线治疗均无效则考虑加用其他免疫抑制剂（霉酚酸酯、硫唑嘌呤、环孢素A、长春新碱和他克莫司等）。对于部分难治或复发患者，可考虑联合血浆置换、免疫吸附及IVIg等方法快速去除血循环中的凝血因子抑制物以达到止血目的[6]。

目前，其他获得性凝血因子缺乏症的特异性治疗指南尚未制订，其治疗方案主要参照AHA患者的IST方案。对于部分难治/复发患者，可考虑血浆置换、免疫吸附及IVIg等方法快速去除血浆中凝血因子抑制物，但疗效无法维持[1],[3]。对于与MGUS相关的AVWD，在抗体无法清除的情况下，在手术中预防出血以及控制急性出血至关重要。对于难治性出血，可尝试使用来那度胺、硼替佐米或达雷妥尤单抗治疗[10]–[11],[50]。

四、总结与展望

与抗凝血因子抗体相关的获得性凝血因子缺乏症较少见，其中以AHA最为常见，其次为AVWD，其他获得性凝血因子缺乏症病例仅有零星报道。由于由不同类别的抗凝血因子抗体引起的凝血因子缺乏症在发病机制、临床表现、诊断、治疗及预后上都有差异，故临床医生需提高对该类疾病的认识，及时正确诊断，有效处理急性出血、积极清除抗体及治疗原发病。近年来，随着更多止血药物的研发，AHA患者止血治疗的疗效及安全性得到了进一步的提高。未来，还需进一步探索针对其他获得性凝血因子缺乏症的止血治疗及抗体清除方案。
